# Compound Fracture of the Elbow-Joint; Removal of the Coronoid Process of the Ulna.—Cured

**Published:** 1838-08-29

**Authors:** J. M. Wallace

**Affiliations:** Late Resident Surgeon


*Compound fracture of the elbow-joint; removal of the coronoid process of the ulna.—Cured.*

[Reported by J. M. Wallace, M. D., late Resident Surgeon.]

Joseph F., aged 45 years, a mason by trade, fell about twenty feet from a scaffolding, upon his right arm, and was brought to the hospital on the 30th of July, 1836. He was found to have an oblique fracture of the olecranon process of the ulna; the upper portion, however, was not drawn up, but was held in its place by the ligamentous attachments; immediately over the fracture there was a wound in the integuments two inches in length, and upon introducing the finger it passed down to the bone, and the fragments could be easily separated. He brought with him a portion of bone, which, he stated, was removed from the wound by a physician who saw him immediately after the injury; it was found to be the entire coronoid process of the ulna; there was no hæmorrhage of any consequence, and but a slight discharge of synovia from the wound; it was dressed with dry lint, the arm was placed upon a rectangular splint, and a loose bandage applied. He was laid upon a fracture-bed in order to keep him perfectly quiet, and cold lotions used over the dressings; low diet.

*August 7th.*—He has suffered but little pain since his admission; the dressings were removed, and suppuration found to be going on; the discharge is of a healthy character; a small poultice applied; allowed soup diet.

*Sept. 3d.*—The wound has been dressed daily without disturbing the arm; he was attacked with erysipelas, which is prevailing extensively through the wards; the limb was covered with cold mucilage, and merely laid in a tin rectangular splint, with a little bran under it, without any bandage.

*Sept. 11th.*—An abscess has formed three inches above the elbow, in consequence of the erysipelas; it was opened, and discharged about two ounces of healthy pus. The wound over the joint was touched with nitrate of silver, and dressed with basilicon ointment; a poultice was applied to the abscess; the fracture of the forearm has united firmly; allowed a bottle of porter and full diet.

*Sept. 17th.*—A carved wooden splint was applied from the axilla to the ends of the fingers, and he was permitted to walk about.

*Sept. 23d.*—The wound over the fracture has closed entirely; the abscess has healed; no motion has been made at the elbow. He went out today on leave of absence and did nct return.

*Nov. 15th.*—He was seen to-day, and found to have motion at the elbow-joint through a quarter of a circle; the motion of the wrist and fingers is perfect, but formation and supination of the hand is very slight. Before this accident he has always enjoyed perfect health, and was strictly temperate in his habits.

